# Evaluation of IR Biotyper for carbapenem-resistant *Pseudomonas aeruginosa* typing and its application potential for the investigation of nosocomial infection

**DOI:** 10.3389/fmicb.2023.1068872

**Published:** 2023-02-09

**Authors:** Yanyan Hu, Kun Zhu, Dingping Jin, Weiyi Shen, Congcong Liu, Hongwei Zhou, Rong Zhang

**Affiliations:** ^1^Clinical Microbiology Laboratory, 2nd Affiliated Hospital of Zhejiang University, School of Medicine, Zhejiang University, Hangzhou, China; ^2^State Key Laboratory of Bioreactor Engineering, East China University of Science and Technology, Shanghai, China; ^3^Infection Control Department, 2nd Affiliated Hospital of Zhejiang University, School of Medicine, Zhejiang University, Hangzhou, China

**Keywords:** carbapenem-resistant *P. aeruginosa*, Fourier-transform infrared spectroscopy, Nosocomial infection investigation, strain typing, WGS-based typing

## Abstract

*Pseudomonas aeruginosa* is one of the most common opportunistic pathogens causing severe nosocomial infections for its patterns of multidrug resistance, particularly for carbapenems. Timely epidemiological surveillance could greatly facilitate infection control of *P. aeruginosa* and many deadly pathogens alike. IR Biotyper (IRBT), is a novel real-time typing tool, based on a Fourier-transform infrared (FTIR) spectroscopy system. It is critical to comprehensively establish and evaluate the feasibility of IRBT in *P. aeruginosa* strain typing. In the current study, we first established standards and schemes for its routine laboratory application, and we found that Mueller–Hinton agar plates give better discriminatory power than blood agar plates. Data showed that the cut-off value of 0.15 with an additional 0.025 range was optimal. Secondly, 27 clinically isolated carbapenem-resistant *P. aeruginosa* (CRPA) strains collected from October 2010 to September 2011 were evaluated for typing effectiveness by comparing IRBT to the other commonly used typing methods, such as multi-locus sequence typing (MLST), pulsed-field gel electrophoresis (PFGE) and whole-genome sequencing (WGS)-based typing. When using WGS-based typing as the reference method, the typing method of FTIR spectroscopy (AR = 0.757, SID = 0.749) could better cluster *P. aeruginosa* strains than MLST and *in silico* serotyping (AR = 0.544, SID = 0.470). Though PFGE showed the highest discriminatory power, low concordance was observed between PFGE and the other methods. Above all, this study demonstrates the utility of the IRBT as a quick, low-cost, real-time typing tool for the detection of CRPA strains.

## Introduction

*Pseudomonas aeruginosa* plays a leading role in infections caused by gram-negative bacilli, especially in critically ill and immunocompromised patients, including ventilator-associated pneumonia, wound infections, and respiratory infections from cystic fibrosis (Lyczak et al., 2000; De Bentzmann and Plesiat, 2011). Due to its properties of biofilms-forming, *P. aeruginosa* can easily survive and spread on the surfaces of hospital equipment and facilities, making it an important source of nosocomial infections and outbreaks (Alipour et al., 2017; Bicking Kinsey et al., 2017). Recently, the increasing incidence of multidrug resistance (MDR), particularly for carbapenems, has further exacerbated *P. aeruginosa* infections and increased mortality (Livermore, 2002; Rossolini and Mantengoli, 2005; Horcajada et al., 2019).

Early detection of cross-transmissions is extremely important in limiting the spread of multidrug-resistant bacterial strains and quickly implementing hygiene control measures to block the spread and minimize the damages. Rapid detection of pathogen cross-transmission in healthcare settings remains challenging, multi-locus sequence typing (MLST), *in silico* serotyping, pulsed-field gel electrophoresis (PFGE), and whole-genome sequencing-based typing (WGS) are several methods that are commonly used at present (Hammer et al., 2003; Waters et al., 2012; Martak et al., 2020),. However, many limitations remain in adapting these methods for regular surveillance, including high costs, labor, time-consuming, and especially the need for professional and technical personnel. Therefore, a convenient, rapid, and reliable bacterial typing technique is urgently required for routine clinical microbiology laboratories.

Fourier transform Infrared Spectroscopy (FT-IR) is an emerging phenotyping technique that is established on a comparison of infrared light absorption spectra patterns and varies according to the whole microorganism cell composition (i.e., lipids, proteins, or polysaccharides). Compared to molecular biology methods, the quick, easy, and low-cost features of FT-IR are highly attractive for routine surveillance (Naumann et al., 1991; Novais et al., 2019). Previous studies of FT-IR typing were commonly based on hardware and manual third-party statistics analysis software. In 2017, the integrated IR Biotyper (IRBT) system that can automatically perform spectra treatment and further comparison by multivariate data analysis tool was launched by Bruker Daltonics. Since then, more research on the potential of FT-IR to differentiate and identify particular groups of strains has been explored in the contexts of clinical or food microbiology (Cordovana et al., 2021; Lombardo et al., 2021; Hu et al., 2021b; Li et al., 2022), particularly for Gram-negative bacteria associated with hospital-acquired infections (Martak et al., 2019).

To our knowledge, no comprehensive estimates of FT-IR typing schemes for clinical *P. aeruginosa* have ever been published. The objectives of the current study are to evaluate the typing effectiveness of clinically isolated carbapenem-resistant *P. aeruginosa* (CRPA) as well as to establish standards and schemes for its routine laboratory application, providing the reference basis for the integration of FT-IR into laboratory epidemiological surveillance and outbreak investigations.

## Materials and methods

### Bacterial isolates

Thirty-eight CRPA isolates were randomly chosen from our previously sequenced genomes from the routine clinical microbiological laboratory of the Second Affiliated Hospital of Zhejiang University from 2010 to 2012. Detailed clinical information is shown in Supplementary Table 1. Before the experiments, all the isolates were re-identified using Matrix-assisted laser desorption/ionization-time of flight mass spectrometry (MALDI-TOF MS) at the species level (Bruker Daltonik GmbH, Bremen, Germany). The selected strains were divided into two groups for the method optimization (*n* = 11) and comparison (*n* = 27) respectively. All the isolates were revived on Columbia blood agar plates (BA; Autobio Diagnostics, Zhengzhou, China) and Mueller–Hinton agar plates (MH) (Oxoid, Wesel, Germany) for 24 ± 2 h at 35°C to optimize the process. MH medium was then chosen for further comparison experiments.

### Pulsed-field gel electrophoresis

PFGE was performed as previously described with slight modification (Hu et al., 2012). Genomic DNAs of the 38 CRPA isolates were digested using the restriction enzyme *Spe*I. *Salmonella enterica* serotype Braenderup H9812 was used as a size marker. A dendrogram based on Dice similarity coefficients was generated from the homology matrix with a coefficient of 0.5% using the unweighted pair group method using arithmetic averages (UPGMA) to describe the relationships among PFGE profiles through the UVIBand software (Bio-Rad). Isolates were considered the same PFGE group if their dice similarity index was ≥80%.

### Genome sequencing

Genomic DNAs of all 38 CRPA isolates were extracted from overnight cultures by using a Wizard genomic DNA purification kit (Promega, Beijing, China) and were subjected to whole-genome sequencing using 150 bp paired-end strategy with the Illumina HiSeq X10 platform (Illumina, San Diego, CA, United States). Raw reads were trimmed and assembled to contigs using SPAdes version 3.11. Acquired antimicrobial resistance genes (ARGs), MLST, and *in silico* serotyping were determined *via* the Center for Genomic Epidemiology website of.^1^ Single-nucleotide polymorphisms (SNPs) were called by the gingr online software of Harvestsuite.^2^ The interpretation of WGS-based typing is more complicated, for which the number of differences depends on the strains collected. Here, we set the allele differences ≤10 as homologous. Previous studies chose a threshold of 14 (Mellmann et al., 2016) or 20 (Martak et al., 2020) alleles of difference for *P. aeruginos*. However, this threshold was not suitable for our study as it would have created too few clusters (data not shown).

### IRBT spectrum acquisition and analysis

An optimal sample preparation method (H_2_O-EtOH) reported in our previous study was applied in the current research (Hu et al., 2021b). Specifically, one inoculation loopful of bacteria, which was grown at 35°C for 24 ± 2 h on BA and MH agar plates, was suspended in 100 μl sterile H_2_O in 1.5 ml vials containing sterile metal rods (Bruker Daltonik, Bremen, Germany), and then 100 μl 70% (vol/vol) ethanol was added after vortex. Subsequently, 15 μl of the homogeneous suspension was taken and spotted onto the 96-well IRBT silicon sample plate (Bruker Daltonik, Bremen, Germany). The sample plate was dried at 37°C for approximately 20 min and four parallel tests were prepared for each sample. The measurements were carried out using an IRBT System (Bruker Daltonik, Bremen, Germany) running the IRBT software (Version 2.0) with the default analysis settings as recommended by the manufacturer. Generally, spectra (4000–500 cm^−1^) of isolates and backgrounds were acquired. For each spectrum, 64 scans were collected in single-beam mode with 4 cm^−1^ resolution at a rate of 3 scans/s. And the spectra range of 1,200–900 cm^−1^ was automatically processed, normalized, and second derivatives by OPUS 7.5 software (Bruker Optics GmbH). Data that did not meet the default quality criteria were excluded in further analysis. All qualified data were analyzed using offline IRBT Client by building dendrograms using the Euclidian distance and average linkage clustering method following the manufacturer’s suggestion.

### Calculation of clustering concordance

The concordance of IRBT clustering in comparison to MLST, *in silico* serotyping, PFGE, and WGS typing methods was determined by calculation of the 95% confidence interval adjusted rand index (Adjusted Rand index, AR) using the online tool.^3^ The Simpson’s index of diversity (SID) was used to evaluate the discriminative ability of each typing method, and the probability of two unrelated isolates separated from the experimental strain set clustering into different typing groups was calculated.

## Results

### Determination of FT-IR clustering cut-off value of *Pseudomonas aeruginosa* typing

**Figure 1:**
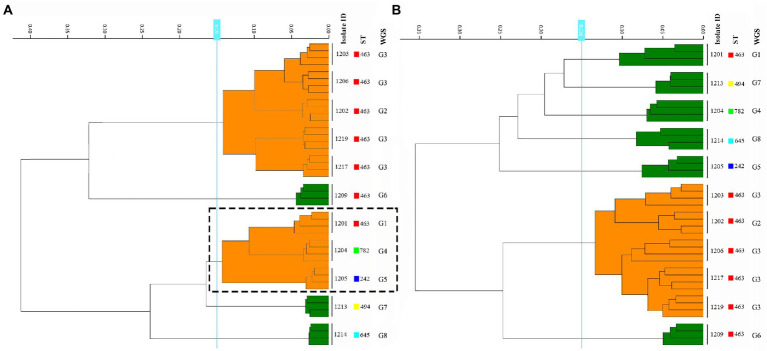
Comparison of the results from the BA and MH agar plate among the 11 *P. aeruginosa* strains. **(A,B)** represent samples prepared in BA and MH medium, respectively. The dashed box showed three distinct ST types that were misclustered together by FT-IR in the BA medium.

The culture medium has been determined before the clustering cut-off value, 11 CRPA isolates of 5 ST types were inoculated on BA and MH agar medium separately and incubated for 24 h ± 2 h. After incubation and spectra acquisition, projects were used to build the dendrograms for each medium using MLST and WGS-based typing data as a reference. As a result of four replicate experiments, albeit the superior growth on the blood agar was to that of MH agar, which was facilitating sample preparation, the failure to correctly discriminate among three distinct genotypes ST463/G1 (1201), ST782/G4 (1204), and ST242/G5 (1205) on BA agar plate (Figure 1; Supplementary Table 2), was considered an “unacceptable error.” Based on this, the MH medium was selected for the subsequent experiments.

**Figure 2:**
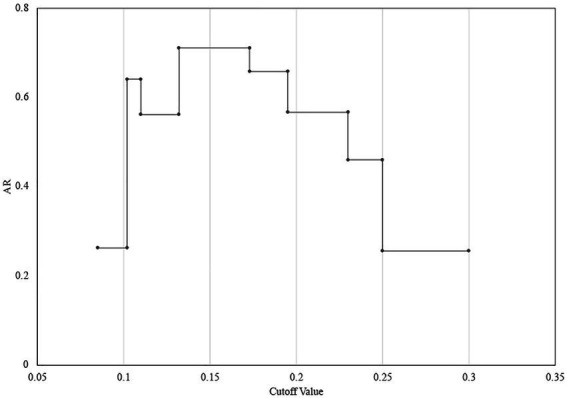
Determination of the FT-IR clustering cut-off value using WGS as the reference. The maximum Adjusted Rand index (AR = 0.711) was reached with a cut-off value range of 0.132–0.173.

The determination of the FT-IR clustering cut-off value was assessed by the concordance of FT-IR types using WGS-based typing as the reference, Adjusted Rand index (AR) was calculated at a different cut-off of clustering (Figure 2). The optimal cut-off varied from 0.132 to 0.173 (AR, 0.711) with one discordance with the WGS-based typing results (Figure 2). Accordingly, 0.15 was used as the COV with an additional 0.025 range in the following experiments.

### Comparison of different typing methods for *Pseudomonas aeruginosa*

**Figure 3:**
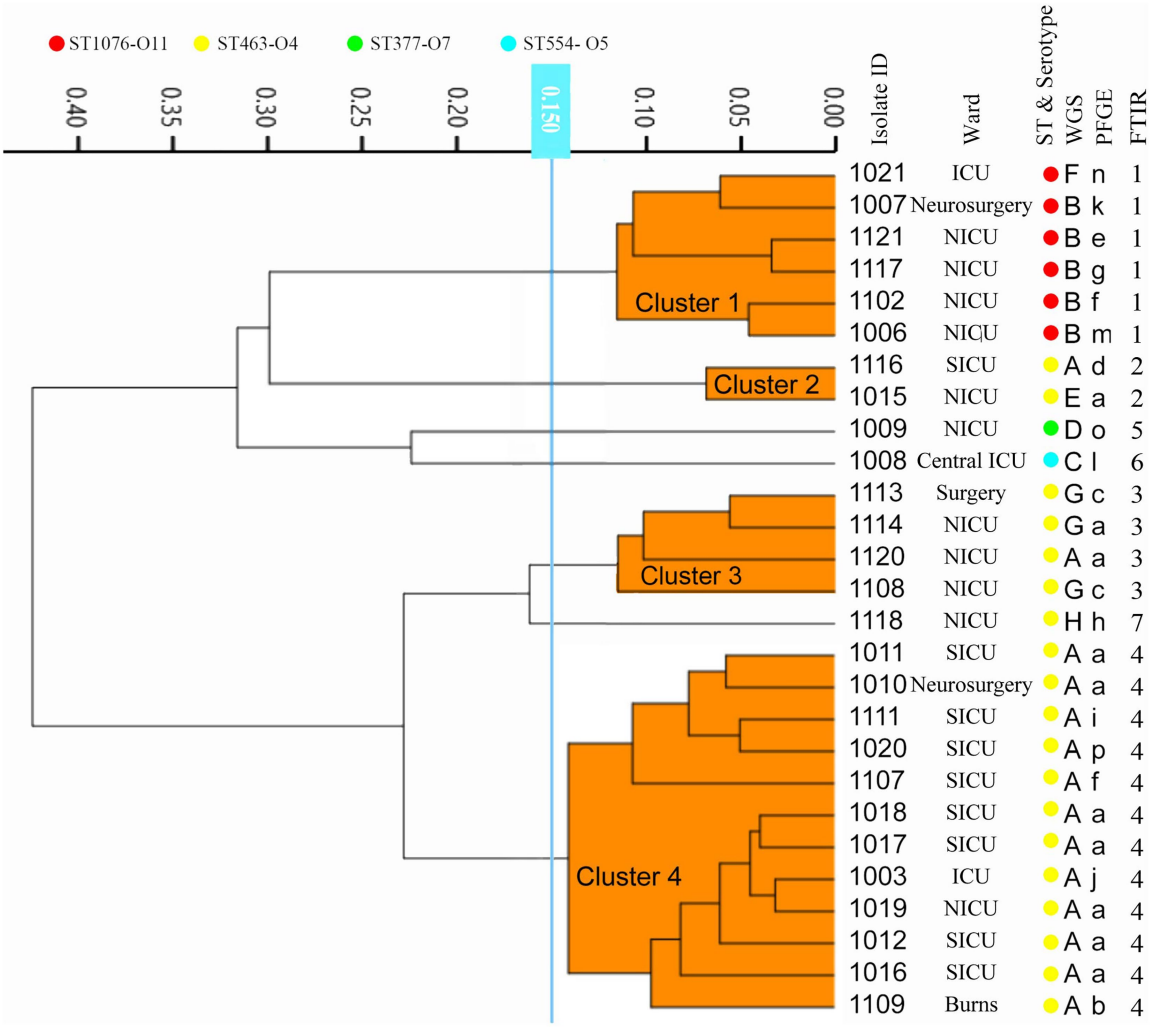
The dendrogram was obtained automatically by FT-IR. The lineages of IRBT, WGS-based typing, PFGE, STs, and serotypes are given for each isolate.

Twenty-seven CRPA isolates collected from different wards over 1 year from October 2010 to September 2011 were analyzed. According to the WGS-based typing results (Figure 3; Supplementary Table 3), 27 isolates could be classified into 8 different types (A-H), with three clusters containing more than one isolate (A, B, G) and an additional 5 singletons. The most predominant type A contained 14 isolates from 5 different locations (SICU, NICU, Neurosurgery, Burns, and ICU). Four MLST types were identified from the 27 isolates, of which ST463 was the dominant ST, which occupied 70.4% (19/27), demonstrating that ST463 was the most prevalent type transmitted within our hospital in the year 2010 and 2011, in combination with its high potential virulence risk had been reported before (Hu et al., 2021a), more awareness should be expected. *In silico* serotyping of these strains was completely congruent with MLST, where serotypes O11, O5, O7, and O4 correspond to ST1076, ST554, ST337, and ST463, respectively. Likewise, O4 was the main serotype. The conventional method PFGE showed a greater discriminatory power (SID = 0.866) by classifying 27 isolates into 16 types (a-p), of which all were singletons except for types a, f, and c (more than 2 isolates). Using the COV range of FT-IR as defined above, the 27 isolates were classified into seven IR types (1–7), four of which contained more than one isolate and the other three were singletons. The concordance analysis of the four methods was calculated through the Adjusted Rand’s index. The highest AR value of 0.757 was obtained between IRBT and WGS, followed by MLST and Serotype typing (AR = 0.544) (Table 1).

**Table 1:** Comparison of different typing methods for CRPA.

Method	No. of types	Simpson’s ID (95% CI)	Adjusted Rand Index				
			MLST	FT-IR	Serotyping	PFGE	WGS
MLST	4	0.470 (0.282–0.659)	–	–	–	–	–
FT-IR	7	0.749 (0.627–0.871)	0.458	–	–	–	–
Serotype	4	0.470 (0.282–0.659)	1.000	0.458	–	–	–
PFGE	16	0.866 (0.754–0.987)	0.230	0.201	0.230	–	–
WGS	8	0.704 (0.543–0.864)	0.544	0.757	0.544	0.245	–

### Evaluation of the capacity for detection of the CRPA outbreak transmission routes

According to the internal 2–1-1 criteria setting for CRPA outbreak identification: two or more CRPAs isolated from the same ward within a month will be identified as a potential nosocomial outbreak and investigation and interventions need to be commenced. By this criteria, five potential outbreaks were included in this study, each from Neurosurgery (1), NICU (2), and SICU (2).

**Figure 4:**
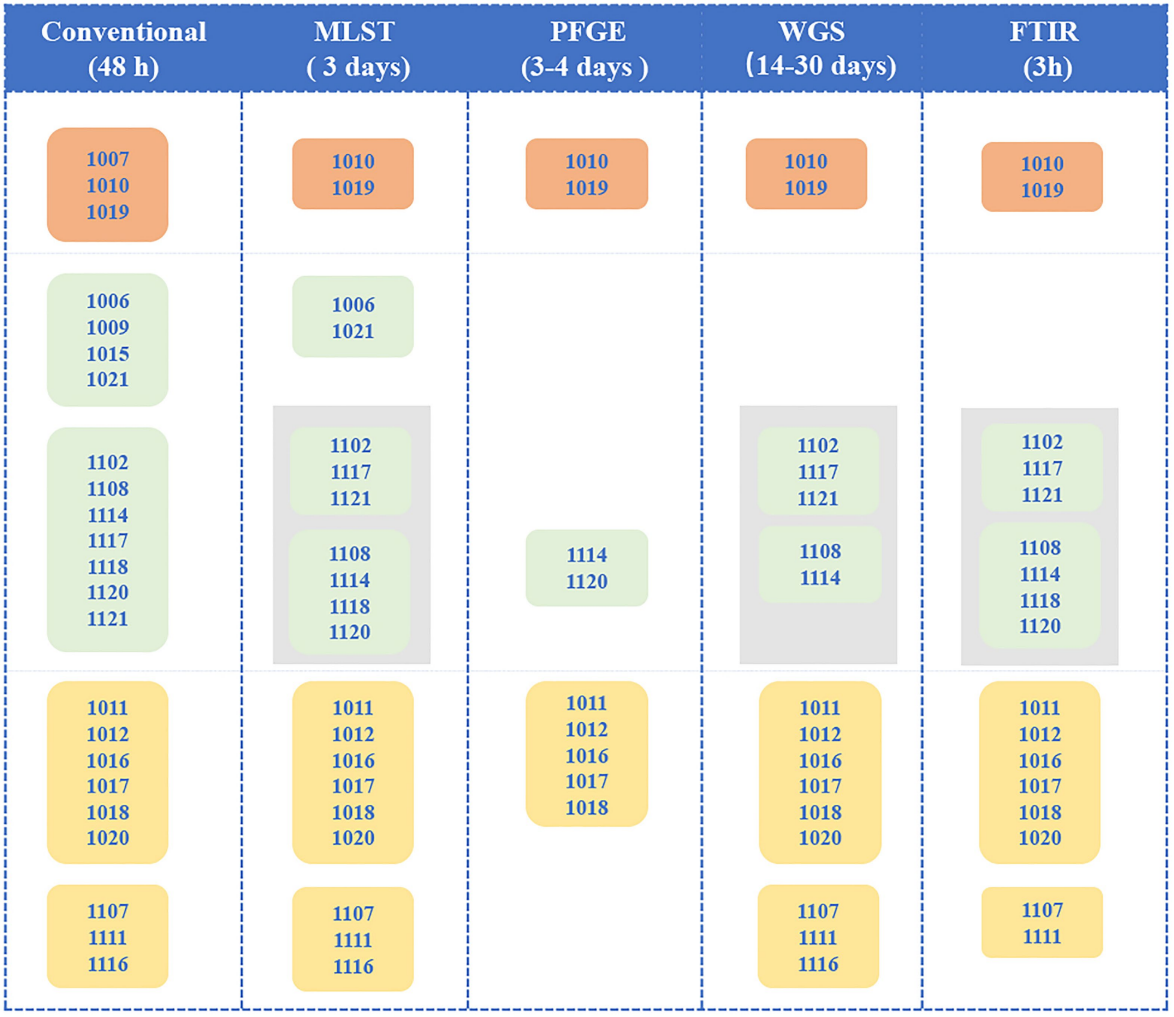
Overview of the capacity of different methods for detection of the CRPA outbreak transmission routes. Different Colors represent outbreaks occurring at different locations: Orange: Neurosurgery; Green: NICU; Yellow: SICU. Each rectangular box represents one outbreak and the number in the box is the relevant isolates ID. Different transmission routes from the same location during the same period are indicated with a gray background. The values in brackets indicate an estimate of the reporting time.

WGS-based typing confirmed four of these nosocomial outbreaks (Figure 4), differing from the conventional results by ruling out one potential outbreak from the NICU, as the four CRPAs isolated in a short period were identified into different subtypes by WGS-based typing. Furthermore, for CRPAs isolated in the NICU from July to September 2011, even though they were isolated from the same location and shared the same drug-resistant phenotype and even resistance genotype (KPC+), the WGS-based typing classified them as different types, which indicates different transmission routes might be involved.

## Discussion

Previous studies have reported a good correlation between commonly used typing methods and FT-IR for different species, showing the ability of FT-IR to decipher the links between epidemiologically-related isolates. However, the COV values automatically allocated by FT-IR systems do not always precisely reflect the sub-species level discrepancies (Rakovitsky et al., 2020), which makes it important to standardize the sample preparation process and the internal reference COV range before formally incorporating IRBT into their laboratory routines.

Accordingly, in this study, with the confirmed cultivation conditions, we first identified internal COV criteria by using the best AR-matching method, using WGS results as references. The 0.15 ± 0.025 range we obtained was comparatively smaller to the unofficial reference from the manufacturer but much smaller than Martak’s results of 0.184–0.374 (Martak et al., 2019), albeit the same optimal AR-matching method was used. The explanation behind this discrepancy may lie in the reference method used in the two studies, the reference method used in Martak’s research was MLST, whereas in the current study more discriminating WGS-based typing had been selected. This further illustrates the significance of the reference method selected when establishing a laboratory’s routine using FT-IR typing parameters and procedure, i.e., the depth of the epidemiological investigation should first be identified. The same discordance is also reflected in the results on *Klebsiella pneumoniae* by the two laboratories, where the variation in reference methods led to differences in the COV (Martak et al., 2019).

According to the comparison of different typing methods, the discriminatory power of FT-IR was higher than that of MLST and phenotypic serotype typing, which is consistent with our previous study (Hu et al., 2021b). PFGE demonstrated the highest discriminatory power in the present study, but its lack of consistency with other methods decreased its reference significance for in-hospital epidemiological investigations. A previous study about *Salmonella enterica* typing methods also showed that strains differed by 0 SNPs presented distinct PFGE subtypes (Kozyreva et al., 2016), suggesting that the distinct PFGE types did not necessarily correlate with increased genetic distance between isolates, PFGE results would be misleading for these isolates, and the relative discriminatory power of different subtyping methods might depend on the strains tested. FT-IR showed the highest concordance with WGS-based typing compared to the other three methods, and the relatively higher resolution allows FT-IR to infer more elaborate genomic linkages or discrepancies than MLST and serotyping, this is indicated by FT-IR can further subdivide the predominantly isolated ST463 into three IR types (2, 3, and 4).

Correctly recognizing outbreaks is the first and most critical step toward effective prevention of in-hospital transmission. This correctness includes finding out the true outbreak and ruling out false positives. In the present study using the WGS as the reference method, 4 out of 5 in-hospital criteria-based outbreaks were confirmed, while the 4 CRPAs from a suspected outbreak in the NICU isolated in 2010 were excluded from relevance. The same results were with PFGE and FT-IR. Due to relatively lower discriminatory power, the same conclusions could not be reached by MLST and *in silico* serotyping. PFGE’s excessively high resolution classified a portion of potentially relevant strains into different types, resulted in a false alert dismissal, and failed to instruct timely and efficient tracking and preventive measures. Besides, we also observed different transmissions occurring in the same location over a short period, such as outbreaks in the NICU in 2011. The presence of two independent transmission routes was detected by multiple methods, giving more detailed evidence for better implementation of corrective interventions.

## Conclusion

As a phenotyping method, our study found that despite standardized schemes for sample preparation, some instability was observed in results when performing a comprehensive analysis of data from different laboratories or batches. Differences in the spectrum caused by operational/system variation will lead to higher COV, further research into the causes and resolutions is required. However, based on the current results, the potential of FT-IR as an epidemiological tool for clinical CRPA is well established. In practice, we recommend the use of FT-IR as a rapid real-time screening option to acquire efficient information for timely preventive and corrective action, the results of which can be further examined and studied using the more sophisticated method like WGS-based typing. This multi-methods program will establish a complete surveillance and investigation system for nosocomial infections.

## Data availability statement

The original contributions presented in the study are included in the article/Supplementary material, further inquiries can be directed to the corresponding author.

## Ethics statement

The studies involving human participants were reviewed and approved by Ethical approval was approved by the Ethics Committee of The Second Affiliated Hospital of Zhejiang University, School of Medicine (Number: 2020–319). Written informed consent for participation was not required for this study in accordance with the national legislation and the institutional requirements.

## Author contributions

RZ and YH: conceptualization and validation. DJ and KZ: methodology. WS, CL, and HZ: formal analysis and data curation. KZ, YH, and RZ: writing, reviewing, and editing. All authors have read and agreed to the published version of the manuscript.

## Conflict of interest

The authors declare that the research was conducted in the absence of any commercial or financial relationships that could be construed as a potential conflict of interest.

## Publisher’s note

All claims expressed in this article are solely those of the authors and do not necessarily represent those of their affiliated organizations, or those of the publisher, the editors and the reviewers. Any product that may be evaluated in this article, or claim that may be made by its manufacturer, is not guaranteed or endorsed by the publisher.

## Footnotes

^1^https://cge.cbs.dtu.dk/services/

^2^http://github.com/marbl/harvest

^3^www.comparingpartitions.info
