# Supplementary material for: Evaluation of IR Biotyper for carbapenem-resistant Pseudomonas aeruginosa typing and its application potential for the investigation of nosocomial infection
Source: Front Microbiol. 2023 Feb 9;14:1068872. doi: 10.3389/fmicb.2023.1068872 (PMC9947493; doi:10.3389/fmicb.2023.1068872)
Supplement: Supplementary file 1 [file Table_1.DOCX]

**Table S1. Clinical information of the *P. aeruginosa* strains used in this study**

| **Isolate ID** | **Isolation Time** | **Age** | **Gender** | **Ward ^*^** | **Sample source** | **KPC** |
| --- | --- | --- | --- | --- | --- | --- |
| 1003 | 2010.10.08 | 90 | Male | ICU | sputum | + |
| 1006 | 2010.10.10 | 69 | Female | NICU | sputum | + |
| 1007 | 2010.10.12 | 61 | Male | Neurosurgery | sputum | + |
| 1008 | 2010.10.13 | 72 | Female | Central ICU | sputum | - |
| 1009 | 2010.10.13 | 70 | Male | NICU | sputum | + |
| 1010 | 2010.10.17 | 57 | Male | Neurosurgery | sputum | + |
| 1011 | 2010.10.20 | 50 | Male | SICU | sputum | + |
| 1012 | 2010.10.21 | 61 | Male | SICU | sputum | + |
| 1015 | 2010.10.25 | 67 | Male | NICU | sputum | - |
| 1016 | 2010.10.25 | 39 | Female | SICU | sputum | + |
| 1017 | 2010.11.02 | 78 | Male | SICU | sputum | + |
| 1018 | 2010.11.02 | 34 | Male | SICU | sputum | + |
| 1019 | 2010.11.05 | 56 | Male | Neurosurgery | cerebrospinal fluid | + |
| 1021 | 2010.11.05 | 25 | Male | NICU | sputum | + |
| 1020 | 2010.11.06 | 58 | Male | SICU | sputum | + |
| 1102 | 2011.07.11 | 33 | Male | NICU | sputum | + |
| 1107 | 2011.07.18 | 62 | Male | SICU | sputum | + |
| 1108 | 2011.07.26 | 35 | Male | NICU | sputum | + |
| 1109 | 2011.07.26 | 41 | Female | Burns | CVC | + |
| 1111 | 2011.07.26 | 52 | Male | SICU | sputum | + |
| 1113 | 2011.08.03 | 59 | Female | Surgery | drainage fluid | + |
| 1114 | 2011.08.03 | 70 | Female | NICU | sputum | + |
| 1116 | 2011.08.10 | 74 | Female | SICU | sputum | + |
| 1117 | 2011.08.22 | 71 | Male | NICU | sputum | + |
| 1118 | 2011.08.27 | 75 | Male | NICU | sputum | + |
| 1120 | 2011.09.03 | 67 | Male | NICU | sputum | + |
| 1121 | 2011.09.06 | 25 | Male | NICU | sputum | + |
| 1201 | 2012.07.22 | 58 | Male | NICU | sputum | - |
| 1202 | 2012.08.04 | 79 | Male | General Surgery | pus | + |
| 1203 | 2012.08.20 | 25 | Female | NICU | sputum | - |
| 1206 | 2012.08.21 | 80 | Female | Healthcare | urine | + |
| 1204 | 2012.08.21 | 60 | Female | NICU | sputum | - |
| 1205 | 2012.08.21 | 29 | Male | EICU | sputum | - |
| 1209 | 2012.09.08 | 28 | Female | Neurology | urine | + |
| 1213 | 2012.10.24 | 83 | Male | Central ICU | sputum | - |
| 1214 | 2012.10.27 | 47 | Male | EICU | sputum | - |
| 1217 | 2012.11.12 | 78 | Female | NICU | sputum | + |
| 1219 | 2012.12.01 | 75 | Male | NICU | sputum | + |

* NICU, Neuro ICU; SICU, Surgery ICU; EICU, Emergency ICU; BICU, Burn ICU

**Table S2** Comparison of different IR typing results under different cultural media conditions with the genotyping methods.

| ID | MLST | WGS  10-SNPs | IR  (BA) | IR  (MH) |
| --- | --- | --- | --- | --- |
| 1201 | 463 | G1 | B3 | M1 |
| 1202 | 463 | G2 | B1 | M6 |
| 1203 | 463 | G3 | B1 | M6 |
| 1204 | 782 | G4 | B3 | M3 |
| 1205 | 242 | G5 | B3 | M5 |
| 1206 | 463 | G3 | B1 | M6 |
| 1209 | 463 | G6 | B2 | M7 |
| 1213 | 494 | G7 | B4 | M2 |
| 1214 | 645 | G8 | B5 | M4 |
| 1217 | 463 | G3 | B1 | M6 |
| 1219 | 463 | G3 | B1 | M6 |

**Table S3. Comparison of different typing methods for *P. aeruginosa* isolates**

| ID | MLST | IR | serotype | PFGE | WGS-10snp | KPC |
| --- | --- | --- | --- | --- | --- | --- |
| 1003 | 463 | 4 | O4 | j | A | + |
| 1006 | 1076 | 1 | O11 | m | B | + |
| 1007 | 1076 | 1 | O11 | k | B | + |
| 1008 | 554 | 6 | O5 | l | C | - |
| 1009 | 377 | 5 | O7 | o | D | + |
| 1010 | 463 | 4 | O4 | a | A | + |
| 1011 | 463 | 4 | O4 | a | A | + |
| 1012 | 463 | 4 | O4 | a | A | + |
| 1015 | 463 | 2 | O4 | a | E | - |
| 1016 | 463 | 4 | O4 | a | A | + |
| 1017 | 463 | 4 | O4 | a | A | + |
| 1018 | 463 | 4 | O4 | a | A | + |
| 1019 | 463 | 4 | O4 | a | A | + |
| 1020 | 463 | 4 | O4 | p | F | + |
| 1021 | 1076 | 1 | O11 | n | A | + |
| 1102 | 1076 | 1 | O11 | f | B | + |
| 1107 | 463 | 4 | O4 | f | A | + |
| 1108 | 463 | 3 | O4 | c | G | + |
| 1109 | 463 | 4 | O4 | b | A | + |
| 1111 | 463 | 4 | O4 | i | A | + |
| 1113 | 463 | 3 | O4 | c | G | + |
| 1114 | 463 | 3 | O4 | a | G | + |
| 1116 | 463 | 2 | O4 | d | A | + |
| 1117 | 1076 | 1 | O11 | g | B | + |
| 1118 | 463 | 7 | O4 | h | H | + |
| 1120 | 463 | 3 | O4 | a | A | + |
| 1121 | 1076 | 1 | O11 | e | B | + |
